# Association between abdominal adiposity and clinical outcomes in patients with acute ischemic stroke

**DOI:** 10.1371/journal.pone.0296833

**Published:** 2024-01-11

**Authors:** Kayo Wakisaka, Ryu Matsuo, Fumi Irie, Yoshinobu Wakisaka, Tetsuro Ago, Masahiro Kamouchi, Takanari Kitazono, on behalf of the Fukuoka Stroke Registry Investigators

**Affiliations:** 1 Department of Medicine and Clinical Science, Graduate School of Medical Sciences, Kyushu University, Fukuoka, Japan; 2 Department of Neurology, Steel Memorial Yawata Hospital, Kitakyushu, Japan; 3 Department of Health Care Administration and Management, Graduate School of Medical Sciences, Kyushu University, Fukuoka, Japan; 4 Center for Cohort Studies, Graduate School of Medical Sciences, Kyushu University, Fukuoka, Japan; Ehime University Graduate School of Medicine, JAPAN

## Abstract

**Background:**

It is unclear whether abdominal adiposity has an additional effect on post-stroke outcomes. This study aimed to determine whether waist circumference (WC) is independently associated with clinical outcomes after acute ischemic stroke.

**Methods:**

We enrolled patients with acute ischemic stroke from a multicenter hospital-based stroke registry in Fukuoka, Japan. We measured WC on admission and categorized patients into four groups (Q1–Q4) according to the quartiles in females and males. The clinical outcomes were poor functional outcome (modified Rankin scale score 2–6) and death from any cause. Logistic regression analysis was performed to estimate the odds ratio and 95% confidence interval of the outcomes of interest after adjusting for potential confounding factors, including body mass index (BMI).

**Results:**

A total of 11,989 patients (70.3±12.2 years, females: 36.1%) were included in the analysis. The risk of poor functional outcome significantly decreased for Q2–Q4 (vs. Q1) at discharge and Q2–Q3 (vs. Q1) at 3 months, even after adjusting for potential confounders, including BMI. In contrast, adjustment of BMI eliminated the significant association between WC and all-cause death at discharge and 3 months. The association between high WC and favorable functional outcome was not affected by fasting insulin levels or homeostatic model assessment for insulin resistance and was only found in patients without diabetes (P = 0.02 for heterogeneity).

**Conclusions:**

These findings suggest that abdominal adiposity has an additional impact on post-stroke functional outcome, independent of body weight and insulin action.

## Introduction

Obesity increases the risk of various diseases such as type 2 diabetes mellitus, cardiovascular diseases, obstructive sleep apnea, and several cancers [1–4]. Conversely, being overweight or obese is also known to paradoxically reduce mortality risk following certain diseases, which is called the “obesity paradox” [5, 6].

Body mass index (BMI) is generally used as an index of overweightness and obesity in studies on the obesity paradox. However, BMI reflects the weight of both adiposity and skeletal muscle, and they probably play distinct roles in functional recovery after stroke. The association between BMI and post-stroke outcomes may be complicated by the combined effects of these factors. Consequently, whether BMI is associated with clinical outcomes in stroke patients is still controversial [7, 8].

Waist circumference (WC) is well correlated with visceral fat mass compared with other anthropometric measures [9, 10]. WC has both independent and additive effects on BMI in various diseases [11]. Accumulation of visceral fat is related to the systemic inflammatory state and insulin resistance [12–14]. Furthermore, visceral adipose tissue plays an important role as an endocrine organ that orchestrates immune and metabolic responses [15, 16]. Since these factors are involved in the recovery from ischemic insult in the brain, WC might be of clinical relevance to outcomes after stroke. However, WC is not routinely measured in clinical practice, and few studies have focused on the association between WC and post-stroke outcomes. Moreover, most studies did not discriminate between WC and BMI, and the findings were inconsistent between the studies [17–20].

Thus, this study aimed to determine whether abdominal adiposity is independently associated with clinical outcomes after ischemic stroke and to elucidate whether this association is mediated by insulin. To address these issues, we measured WC upon admission in patients with acute ischemic stroke and investigated the association between WC and post-stroke clinical outcomes using a multicenter hospital-based stroke registry in Japan.

## Materials and methods

### Study design

The Fukuoka Stroke Registry (FSR) is a multicenter hospital-based registry of Japanese patients with acute stroke hospitalized at seven stroke centers in Fukuoka Prefecture, southern Japan. The FSR enrolled consecutive patients with acute stroke within seven days of onset (UMIN Clinical Trial Registry, UMIN000000800). The participating hospitals were Kyushu University Hospital (Fukuoka, Japan), National Hospital Organization Kyushu Medical Center (Fukuoka, Japan), National Hospital Organization Fukuoka–Higashi Medical Center (Koga, Japan), Fukuoka Red Cross Hospital (Fukuoka, Japan), St. Mary’s Hospital (Kurume, Japan), Steel Memorial Yawata Hospital (Kitakyushu, Japan), and Japan Labour Health and Welfare Organization Kyushu Rosai Hospital (Kitakyushu, Japan). The Institutional Review Boards of all participating hospitals approved the study design of the FSR (approval numbers, Kyushu University Institutional Review Board for Clinical Research: 22086–01, Kyushu Medical Center Institutional Review Board: R06-03, Clinical Research Review Board of Fukuokahigashi Medical Center: 29-C-38, Fukuoka Red Cross Hospital Institutional Review Board: 629, St. Mary’s Hospital Research Ethics Review Committee: S13-0110, Steel Memorial Yawata Hospital Ethics Committee: 06-04-13, and Kyushu Rosai Hospital Institutional Review Board: 21–8). Written informed consent was obtained from all patients or their family members. Stroke was defined as the sudden onset of non-convulsive and focal neurological deficits. Ischemic stroke was diagnosed using computed tomography, magnetic resonance imaging, or both.

### Study patients

Altogether, 15,569 patients with acute ischemic stroke were enrolled in a prospective FSR study from June 2007 to September 2019. We excluded patients who were functionally dependent before admission (modified Rankin scale [mRS] score ≥2) to avoid the influence of functional status before stroke onset [21, 22]. Consequently, 3,386 patients were excluded from the analysis. Another 194 patients lacking data on the variables for multivariable analysis were also excluded. Finally, we included 11,749 patients in the main analysis after excluding 240 patients who were lost during the follow-up period of 3 months. S1 Fig depicts the patient selection flowchart.

### Waist circumference

WC was measured on admission by trained nurses at the level of the largest abdominal circumference with the patient in the supine position. We categorized WC into four groups according to quartiles in each sex: Q1 (females: ≤74.3 cm, males: ≤78.9 cm), Q2 (females: 74.5–81.8 cm, males: 79.0–84.9 cm), Q3 (females: 82.0–88.8 cm, males: 85.0–90.8 cm), and Q4 (females: ≥89.0 cm, males: ≥91.0 cm).

### Clinical variables

We assessed clinical variables such as demographics, risk factors, pre-stroke functional status, previous stroke, stroke subtype, neurological severity, and reperfusion therapy. The risk factors included hypertension, diabetes mellitus, dyslipidemia, and atrial fibrillation. Pre-stroke functional status was assessed using the mRS (mRS score 0 or 1) before onset [21, 22]. Previous strokes included ischemic and hemorrhagic strokes before the onset of the index stroke. Stroke subtypes were categorized into cardioembolism, small-vessel occlusion, large-artery atherosclerosis, and others (stroke of other determined etiology and stroke of undetermined etiology) according to the TOAST classification [23]. Neurological severity was evaluated on admission using the National Institutes of Health Stroke Scale (NIHSS). Reperfusion therapy included the use of thrombolytic agents such as recombinant tissue plasminogen activator, mechanical thrombectomy, or both.

### BMI and insulin action

Body weight was assessed on admission. BMI was calculated by dividing weight in kilograms by height in meters squared. To evaluate insulin action, we measured plasma glucose and insulin concentrations during the fasting state. Fasting insulin levels were measured using plasma immunoreactive insulin (IRI). To estimate pancreatic β-cell function and insulin resistance, we calculated homeostatic model assessment of beta cell function (HOMA-β) and homeostatic model assessment for insulin resistance (HOMA-IR) using the following formulae: HOMA-β, fasting IRI (mU/L) × 360 / (fasting blood glucose [mg/dL] - 63); HOMA-IR, fasting blood glucose (mg/dL) × fasting IRI (mU/L) / 405.

### Study outcomes

The study outcomes were poor functional outcome (mRS score 2–6) and death from any cause (mRS score 6) 3 months after onset. In the sensitivity analysis, the outcomes were assessed at discharge to avoid the influence of the intervention during the post-acute stage. Additionally, functional dependency (mRS score 2–5) was evaluated as another functional outcome to exclude the influence of death on functional status. Stroke neurologists evaluated the study outcomes at discharge. A follow-up study at 3 months was performed by well-trained authorized nurses using a standardized questionnaire in person or by telephone.

### Statistical analysis

Baseline characteristics were compared among WC groups using the Kruskal–Wallis test for continuous variables and the χ squared test for categorical variables. Trends in baseline data were also assessed according to WC groups using the Jonckheere–Terpstra test for continuous variables and the Cochran–Armitage trend test for categorical variables. Logistic regression analysis was performed to estimate the odds ratio (OR) and 95% confidence interval (CI) of each outcome of interest after adjusting for covariates. The multivariable model included age, sex, risk factors (hypertension, diabetes mellitus, dyslipidemia, and atrial fibrillation), pre-stroke functional status (mRS score of 0 or 1 before onset), previous history of stroke, stroke subtype (cardioembolism, small-vessel occlusion, large-artery atherosclerosis, or others), NIHSS score on admission, and reperfusion therapy. BMI and parameters of insulin action, such as IRI, HOMA-β, and HOMA-IR, were additionally included in the multivariable model when needed. Model fitting for clinical outcomes after stroke was evaluated using the Akaike and Bayesian information criteria. The difference in fit between the two models was tested using the likelihood ratio.

We performed subgroup analysis in the groups stratified by the ability of insulin secretion (HOMA-β: ≤30 or >30) and insulin sensitivity (HOMA-IR: <2.5 or ≥2.5). Subgroup analysis was also performed in the groups stratified by age (<65 or ≥65 years), sex, diabetes mellitus, or BMI (<23 or ≥23), which potentially confound the association between WC and post-stroke outcomes. Heterogeneity was evaluated using the likelihood ratio test after adding an interaction term for WC categories × subgroups.

In the sensitivity analysis, all the hospitalized 11,989 patients were included to evaluate the association with discharge outcomes.

Two-tailed P values <0.05 were considered statistically significant. Statistical analyses were performed using STATA version 16.0 (StataCorp LLC, College Station, TX, USA).

## Results

### Baseline characteristics

In all, 11,749 patients (70.3±12.2 years, females: 36.1%) were included in the analysis for 3-month outcomes. Patients were younger and the prevalence of hypertension, diabetes mellitus, dyslipidemia, and no disability before onset (mRS score 0) increased as WC increased, whereas atrial fibrillation was less frequent with increasing WC (Table 1). The proportion of cardioembolisms decreased as the WC increased. Neurological symptoms were less severe and reperfusion therapy was less frequent with increasing WC. The BMI, fasting insulin levels, HOMA-β, and HOMA-IR values increased as the WC increased.

**Table 1:** Baseline characteristics of patients according to waist circumference.

	Q1	Q2	Q3	Q4	P	P_trend_
	n = 2736	n = 2885	n = 3002	n = 3126		
Age, y	72±13	71±12	70±11	68±12	<0.001	<0.001
Males	1684 (61.5)	1858 (64.4)	1951 (65.0)	2014 (64.4)	0.03	0.02
Risk factors						
Hypertension	1931 (70.6)	2236 (77.5)	2505 (83.4)	2736 (87.5)	<0.001	<0.001
Diabetes mellitus	585 (21.4)	821 (28.5)	954 (31.8)	1237 (39.6)	<0.001	<0.001
Dyslipidemia	1137 (41.6)	1584 (54.9)	1871 (62.3)	2166 (69.3)	<0.001	<0.001
Atrial fibrillation	745 (27.2)	611 (21.2)	593 (19.8)	603 (19.3)	<0.001	<0.001
Pre-stroke mRS 1	385 (14.1)	347 (12.0)	360 (12.0)	335 (10.7)	0.001	<0.001
Previous stroke	390 (14.3)	452 (15.7)	496 (16.5)	468 (15.0)	0.10	0.36
Stroke subtype						
Cardioembolism	696 (25.4)	532 (18.4)	514 (17.1)	494 (15.8)	<0.001	<0.001
Small-vessel occlusion	685 (25.0)	887 (30.7)	914 (30.4)	998 (31.9)	<0.001	<0.001
Large-artery atherosclerosis	390 (14.3)	466 (16.2)	495 (16.5)	529 (16.9)	0.03	0.007
Unclassified	965 (35.3)	1000 (34.7)	1079 (35.9)	1105 (35.3)	0.79	0.70
Baseline NIHSS score	3 (1–6)	2 (1–5)	2 (1–4)	2 (1–4)	<0.001	<0.001
Reperfusion therapy	331 (12.1)	298 (10.3)	307 (10.2)	304 (9.7)	0.02	0.005
BMI	19.8±2.3	22.1±2.0	23.8±2.1	27.0±3.4	<0.001	<0.001
Insulin action						
IRI, mU/L	4.5 (3.0–6.9)	5.5 (3.8–8.4)	6.7 (4.6–10.2)	8.7 (5.8–12.7)	<0.001	<0.001
HOMA-β	46 (30–70)	54 (33–82)	61 (38–94)	70 (43–109)	<0.001	<0.001
HOMA-IR	1.1 (0.7–1.9)	1.4 (1.0–2.3)	1.8 (1.1–2.9)	2.3 (1.5–3.7)	<0.001	<0.001

P_trend_, P for trend; SD, standard deviation; mRS, modified Rankin scale; NIHSS, National Institutes of Health Stroke Scale; IQR, interquartile range; BMI, body mass index; IRI, immunoreactive insulin; HOMA-β, homeostatic model assessment of beta cell function; HOMA-IR, homeostatic model assessment of insulin resistance.

^a^Data are expressed as mean±SD, n (%), or median (interquartile range).

^b^Waist circumference was categorized into four groups according to quartiles in females (Q1: ≤74.3 cm, Q2: 74.5–81.8 cm, Q3: 82.0–88.8 cm, and Q4: ≥89.0 cm) and males (Q1: ≤78.9 cm, Q2: 79.0–84.9 cm, Q3: 85.0–90.8 cm, and Q4: ≥91.0 cm).

### Association between WC and clinical outcomes

First, we evaluated the association between WC and poor functional outcome at 3 months (Table 2). The proportion of poor functional outcome decreased as WC increased, and the ORs of poor functional outcome showed a decreasing trend from Q2 to Q4 vs. Q1 after adjusting for potential confounding factors, except BMI. Even after further adjustment for BMI, the ORs of poor functional outcome were still significantly lower for Q2–Q3 compared with Q1. However, the linear trend disappeared and the relationship was L-shaped.

**Table 2:** Association between waist circumference and poor functional outcome.

		Age and sex-adjusted		MV-adjusted		MV and BMI-adjusted*	
	Events	OR	P	OR	P	OR	P
	n (%)	(95% CI)		(95% CI)		(95% CI)	
Q1, n = 2736	1158 (42.3)	1.00 (reference)		1.00 (reference)		1.00 (reference)	
Q2, n = 2885	965 (33.4)	0.73 (0.65–0.82)	<0.001	0.77 (0.68–0.88)	<0.001	0.81 (0.71–0.93)	0.003
Q3, n = 3002	917 (30.5)	0.65 (0.58–0.73)	<0.001	0.70 (0.61–0.80)	<0.001	0.76 (0.66–0.89)	<0.001
Q4, n = 3126	927 (29.7)	0.68 (0.60–0.76)	<0.001	0.71 (0.62–0.82)	<0.001	0.83 (0.69–1.00)	0.05
P for trend			<0.001		<0.001		0.62

MV, multivariable; BMI, body mass index; OR, odds ratio; CI, confidence interval.

^a^Waist circumference was categorized into four groups according to quartiles in females (Q1: ≤74.3 cm, Q2: 74.5–81.8 cm, Q3: 82.0–88.8 cm, and Q4: ≥89.0 cm) and males (Q1: ≤78.9 cm, Q2: 79.0–84.9 cm, Q3: 85.0–90.8 cm, and Q4: ≥91.0 cm).

^b^The multivariable model included age, sex, hypertension, diabetes mellitus, dyslipidemia, atrial fibrillation, pre-stroke modified Rankin scale score, history of stroke, stroke subtype (cardioembolism, small-vessel occlusion, large-artery atherosclerosis, or others), National Institutes of Health Stroke Scale score on admission, and reperfusion therapy.

*BMI added to multivariable model.

Next, we investigated whether WC was associated with the risk of death within three months of onset. Crude death rates decreased as WC increased (Table 3). Although a decreasing trend in death was still found according to WC even after adjusting for confounders except BMI, the additional inclusion of BMI in the multivariable model eliminated the significant association.

**Table 3:** Association between waist circumference and death.

		Age and sex-adjusted		MV-adjusted		MV and BMI-adjusted*	
	Events	OR	P	OR	P	OR	P
	n (%)	(95% CI)		(95% CI)		(95% CI)	
Q1, n = 2736	97 (3.5)	1.00 (reference)		1.00 (reference)		1.00 (reference)	
Q2, n = 2885	65 (2.3)	0.70 (0.51–0.96)	0.03	0.81 (0.58–1.14)	0.23	1.11 (0.77–1.61)	0.56
Q3, n = 3002	41 (1.4)	0.44 (0.30–0.63)	<0.001	0.52 (0.35–0.76)	<0.001	0.87 (0.55–1.38)	0.56
Q4, n = 3126	35 (1.1)	0.39 (0.26–0.58)	<0.001	0.49 (0.32–0.74)	<0.001	1.18 (0.66–2.08)	0.58
P for trend			<0.001		<0.001		0.28

MV, multivariable; BMI, body mass index; OR, odds ratio; CI, confidence interval.

^a^Waist circumference was categorized into four groups according to quartiles in females (Q1: ≤74.3 cm, Q2: 74.5–81.8 cm, Q3: 82.0–88.8 cm, and Q4: ≥89.0 cm) and males (Q1: ≤78.9 cm, Q2: 79.0–84.9 cm, Q3: 85.0–90.8 cm, and Q4: ≥91.0 cm).

^b^The multivariable model included age, sex, hypertension, diabetes mellitus, dyslipidemia, atrial fibrillation, pre-stroke modified Rankin scale score, history of stroke, stroke subtype (cardioembolism, small-vessel occlusion, large-artery atherosclerosis, or others), National Institutes of Health Stroke Scale score on admission, and reperfusion therapy.

*BMI added to multivariable model.

We evaluated how model fit is improved by adding BMI or WC to the multivariable model including WC or BMI, respectively (S1 Table). Consequently, the addition of WC or BMI to the multivariable model improved the model fit for poor functional outcome. The addition of BMI to the multivariable model improved the model fit for the risk of death; however, the model fit was not improved by the addition of WC.

### Association between WC and functional outcomes in relation to insulin action

To explore whether the association between WC and 3-month poor functional outcome is mediated by insulin action, we examined whether the association was altered by additional adjustments for various parameters of insulin action in patients who had not been treated with insulin before stroke onset or did not receive insulin therapy during hospitalization for index stroke. Consequently, the associations of WC with clinical outcomes were unchanged even after additionally adjusting for fasting IRI, HOMA-β, or HOMA-IR (Table 4). No heterogeneity was found in the associations according to HOMA-β or HOMA-IR levels (S2 Table).

**Table 4:** Association between waist circumference and clinical outcomes in consideration of insulin action.

		MV, BMI, and IRI-adjusted*		MV, BMI, and HOMA-β-adjusted*		MV, BMI, and HOMA-IR-adjusted*	
	Events	OR	P	OR	P	OR	P
	n (%)	(95% CI)		(95% CI)		(95% CI)	
Poor functional outcome							
Q1, n = 2345	943 (40.2)	1.00 (reference)		1.00 (reference)		1.00 (reference)	
Q2, n = 2406	732 (30.4)	0.81 (0.69–0.95)	0.01	0.80 (0.68–0.94)	0.008	0.80 (0.68–0.94)	0.007
Q3, n = 2499	686 (27.5)	0.73 (0.61–0.87)	0.001	0.73 (0.61–0.87)	0.001	0.73 (0.60–0.87)	0.001
Q4, n = 2466	649 (26.3)	0.80 (0.64–1.00)	0.06	0.81 (0.65–1.01)	0.06	0.80 (0.64–0.99)	0.045
P for trend			0.58		0.67		0.53
Death							
Q1, n = 2345	69 (2.9)	1.00 (reference)		1.00 (reference)		1.00 (reference)	
Q2, n = 2406	48 (2.0)	1.26 (0.77–2.05)	0.36	1.31 (0.80–2.13)	0.28	1.25 (0.77–2.04)	0.36
Q3, n = 2499	27 (1.1)	0.90 (0.48–1.69)	0.74	0.95 (0.51–1.80)	0.89	0.90 (0.48–1.70)	0.75
Q4, n = 2466	24 (1.0)	1.50 (0.71–3.19)	0.29	1.61 (0.75–3.44)	0.22	1.48 (0.70–3.15)	0.31
P for trend			0.30		0.21		0.30

MV, multivariable; BMI, body mass index; IRI, immunoreactive insulin; HOMA-β, homeostatic model assessment of beta cell function; HOMA-IR, homeostatic model assessment for insulin resistance; OR, odds ratio; CI, confidence interval.

^a^Waist circumference was categorized into four groups according to quartiles in females (Q1: ≤74.3 cm, Q2: 74.5–81.8 cm, Q3: 82.0–88.8 cm, and Q4: ≥89.0 cm) and males (Q1: ≤78.9 cm, Q2: 79.0–84.9 cm, Q3: 85.0–90.8 cm, and Q4: ≥91.0 cm).

^b^The multivariable model included age, sex, hypertension, diabetes mellitus, dyslipidemia, atrial fibrillation, pre-stroke modified Rankin scale score, history of stroke, stroke subtype (cardioembolism, small-vessel occlusion, large-artery atherosclerosis, or others), National Institutes of Health Stroke Scale score on admission, and reperfusion therapy.

*BMI and IRI, HOMA-β, or HOMA-IR were added to the multivariable model.

### Subgroup analysis

**Fig 1:**
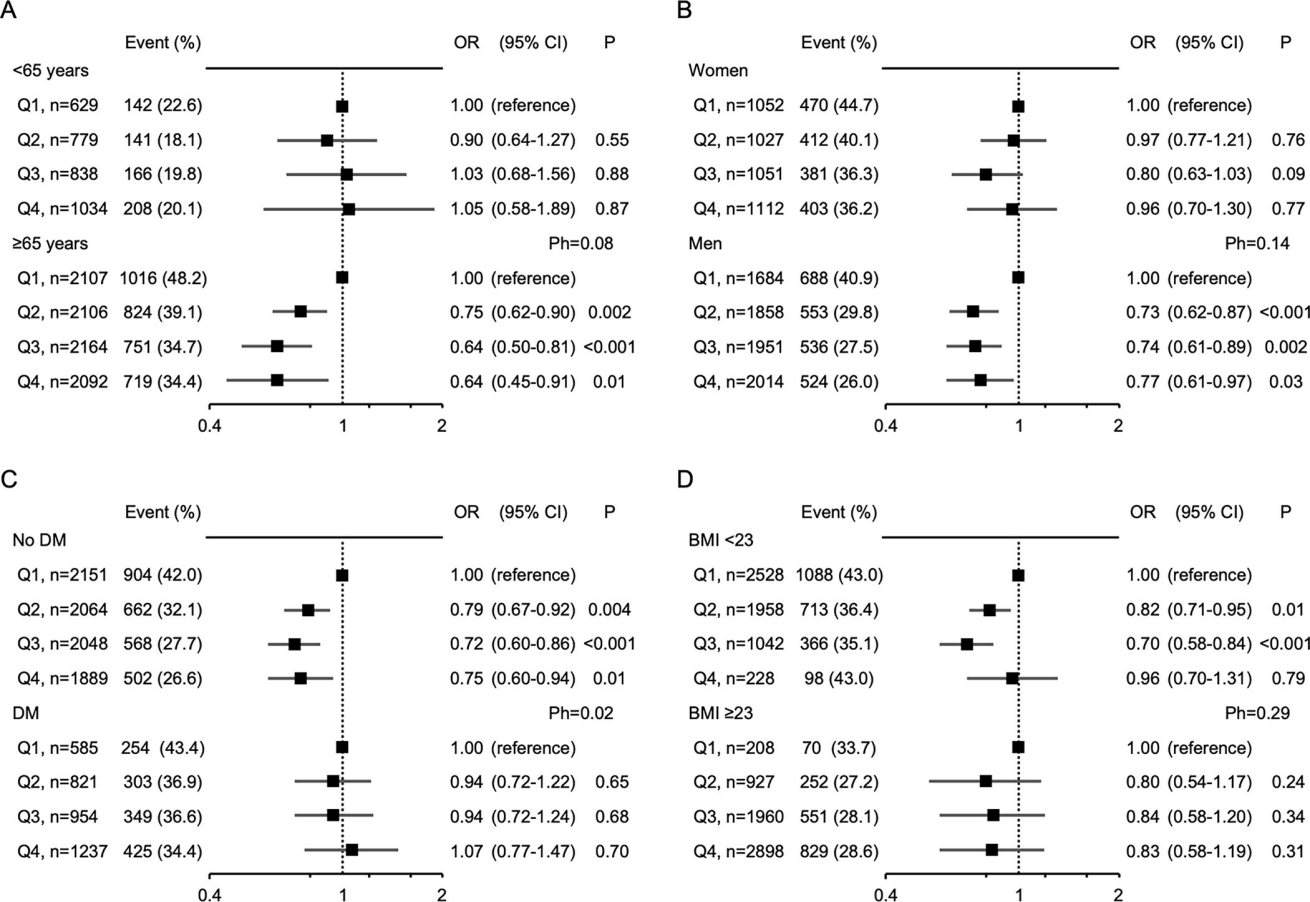
Subgroup analysis for the association between WC and poor functional outcome at 3 months according to age, sex, diabetes mellitus, and obesity. OR, odds ratio; CI, confidence interval; Ph, P for heterogeneity; DM, diabetes mellitus; BMI, body mass index; WC, waist circumference. The association between WC and poor functional outcome at 3 months is shown separately in subgroups according to age (A, <65 years and ≥65 years), sex (B, females and males), diabetes mellitus (C, non-diabetes mellitus and diabetes mellitus), and obesity (D, BMI <23 and BMI ≥23). Waist circumference was categorized into four groups according to quartiles in females (Q1: ≤74.3 cm, Q2: 74.5–81.8 cm, Q3: 82.0–88.8 cm, and Q4: ≥89.0 cm) and males (Q1: ≤78.9 cm, Q2: 79.0–84.9 cm, Q3: 85.0–90.8 cm, and Q4: ≥91.0 cm). Poor functional outcome was defined as a modified Rankin scale score of 2–6. The multivariable model included age, sex, hypertension, diabetes mellitus, dyslipidemia, atrial fibrillation, pre-stroke modified Rankin scale score, previous history of stroke, stroke subtype (cardioembolism, small-vessel occlusion, large-artery atherosclerosis, or others), National Institutes of Health Stroke Scale score on admission, reperfusion therapy, and BMI.

Thereafter, we performed a subgroup analysis to determine whether effect modification was found in the association between WC and 3-month poor functional outcome by specific factors, such as age, sex, diabetes mellitus, and obesity. Consequently, the association was significant in older patients, male patients, patients without diabetes, or patients who were not obese (Fig 1). Among these factors, heterogeneity was statistically significant according to diabetes mellitus status. High WC was associated with a low risk of poor functional outcome only in patients without diabetes.

### Sensitivity analysis

Finally, we conducted two types of sensitivity analyses. To exclude the possible influence of interventions in the post-subacute stage, we investigated the association between WC and clinical outcomes at discharge in 11,989 patients (70.3±12.2 years, females: 36.1%, S3 Table). Similar associations were observed in the discharge outcome cohort (S4 Table).

We also investigated the association between WC and functional dependency (mRS score 2–5) in survivors to exclude the influence of death on functional status. Consequently, a high WC was still associated with a low risk of functional dependency at discharge and at 3 months (S5 Table), which was not affected by insulin action (S6 Table).

The addition of WC to the multivariable model, including BMI, improved the model fit for functional outcomes at discharge but not for in-hospital death, whereas the addition of BMI to the model including WC showed the opposite results (S7 Table). The subgroup analysis showed the same trends in the association between WC and functional dependency (S2 Fig).

## Discussion

The major findings of this study are as follows: (1) High WC was independently associated with favorable functional outcomes apart from BMI in patients with acute ischemic stroke, and the relationship was not linear but L-shaped; (2) In contrast to functional outcomes, WC was not associated with post-stroke death after adjusting for BMI; (3) An effect modification was found in diabetes mellitus; the association was stronger in patients without diabetes than in patients with diabetes; and (4) The association between high WC and favorable functional outcomes was maintained regardless of fasting insulin levels, insulin secretory ability, and insulin sensitivity. These findings suggest that WC has independent and additional effects on functional outcomes, but not on death, in patients with acute ischemic stroke.

### Obesity paradox in stroke patients

BMI is generally used as the gold standard index for overweightness and obesity. Many studies have investigated the obesity paradox using BMI in patients with stroke, though their findings were inconsistent [24–26]. BMI represents the total mass of both skeletal muscle and adipose tissue; however, they probably play different roles in recovery after stroke. Moreover, the characteristics and functional roles in metabolic regulation differ substantially between subcutaneous and visceral adipose tissues [27–29]. Therefore, the impact of BMI on post-stroke outcomes may differ according to the proportion of these compartments. A previous systematic review raised the issue that most studies investigated the relationship between WC and morbidity or mortality without adjusting for BMI [30]. As WC represents the sum of abdominal subcutaneous fat mass and visceral fat mass, it may be a better marker than BMI for investigating the association between abdominal adiposity and post-stroke outcomes. The present study clearly indicated that WC is an independent factor for post-stroke outcomes even after taking BMI into account.

### Association between abdominal adiposity and post-stroke outcomes

In the present study, favorable functional outcomes and survival rates increased with increasing WC. However, adjustment for BMI resulted in a substantial difference in the association depending on the outcomes.

High WC showed a significant association with favorable functional outcomes, whereas the significant association between WC and death disappeared. Given that its association with WC is partly mediated by BMI, post-stroke survival may be affected by certain components of BMI. A population-based study demonstrated that the appendicular skeletal muscle mass index (ASMI) was positively associated with BMI regardless of total body fat and that ASMI was independently associated with a reduced risk of mortality [31]. From our findings, abdominal fat may be less important for post-stroke survival than the other components of BMI.

In contrast to the risk of death, a high WC was significantly associated with favorable functional outcomes, even after adjusting for BMI. Our findings are contradictory to a previous study that reported that low visceral abdominal fat proportion was associated with favorable and excellent outcomes after intravenous thrombolysis for acute ischemic stroke [32]. Nevertheless, in our study, the linear relationship between WC and functional outcomes became L-shaped after controlling for BMI. This phenomenon implies that “the fatter, the better” theory is not applicable to abdominal fat in terms of post-stroke functional status.

### Populations susceptible to abdominal adiposity

Whether sex differences exist in the obesity paradox in stroke patients remains unclear. A favorable association of high WC was found in males in a Korean study [18] but in females in a Polish study [19]. In our study, a favorable association between WC and post-stroke functional status was obvious in males. Females have higher amounts of subcutaneous adipose tissue and less visceral adipose tissue than males [33, 34]. This disproportional distribution of fat between sexes may cause sex differences. Additional studies are required to elucidate the presence of sex differences across ethnic groups because genetic factors cause variations in fat distribution [34, 35].

The present study also demonstrated a significant association between older patients, patients without diabetes, and lean patients. A common characteristic of these patients may be low fat mass. The effect modification was statistically significant according to diabetes mellitus status. An association between WC and unfavorable functional outcomes was found only in patients without diabetes mellitus. Therefore, patients without diabetes who are unable to accumulate abdominal fat are at an increased risk of poor functional recovery after acute ischemic stroke.

### Putative mechanisms

The reason for high WC showing a favorable relationship to functional outcomes after stroke is unclear. Our previous study demonstrated that insulin action plays an important role in functional outcomes after ischemic stroke [36, 37]. Since high WC potentially causes insulin resistance, we hypothesized that insulin action may affect these associations [36, 37]. High BMI was associated with favorable outcomes only in insulin-resistant patients in a Chinese study [38]. However, in our study, the favorable association between WC and post-stroke functional outcomes was not altered by fasting insulin levels, pancreatic β-cell function, or insulin sensitivity. Our findings suggest that abdominal adiposity is related to post-stroke functional outcomes through a pathway distinct from that of insulin.

One plausible explanation is that abdominal fat plays a key role as an energy reservoir in critically ill patients. After the onset of stroke, metabolic and catabolic states are unbalanced because of abnormalities in neuroendocrine activation and accumulation of cytokines and oxygen-free radicals, leading to the loss of both fat and muscle tissue [39]. The sympathetic neuroendocrine system is stimulated after stroke, which contributes to catabolic activation [39]. Besides catecholamines, natriuretic peptide, cortisol, and corticotropin are increased after stroke, leading to an overall catabolic dominance [39]. In lean patients, skeletal muscle mass decreases via catabolization in addition to immobility during hospitalization after stroke because fat mass is scarce. In contrast, patients with abundant abdominal fat may have sufficient metabolic reserves to endure fat consumption [40], which may cause functional restoration after stroke.

Another possibility is that abdominal adiposity facilitates functional recovery after acute ischemic stroke. Subcutaneous and visceral adipose tissues may mediate different adipogenic and immunomodulatory functions [27, 29]. Recent studies have revealed that visceral adipose tissue plays a key role in linking the inflammatory state to the metabolic state as an endocrine organ [13, 16]. In animal models of ischemic stroke, cytokines were upregulated, which triggered proinflammatory (e.g., interleukin [IL]-1β, tumor necrosis factor [TNF], and IL-6) and anti-inflammatory (transforming growth factor-β and IL-10) mechanisms [41]. A recent study in patients with ischemic stroke reported that BMI was inversely associated with IL-6 levels after major stroke, while eotaxin, IFN-β, IFN-γ, and TNF-α were upregulated when BMI increased [42]. These inflammatory cytokines potentially have neurotoxic and neuroprotective effects according to the phase after stroke onset [43]. Post-stroke inflammation in the brain and its modulation with adipose tissue may play pivotal roles in the association between WC and functional outcome after ischemic stroke.

The present study suggested that baseline WC on admission was associated with 3-month functional status. A previous study reported that fat loss started from the day of admission in patients with ischemic stroke [42]. Therefore, loss of fat after the onset may have an additional impact on functional outcome in patients with ischemic stroke. Furthermore, moderate weight loss and cachexia were found even 1 year after onset in a considerable proportion of patients with stroke [44]. Based on these findings, suitable nutrition and weight management in the post-acute as well as chronic stages might be crucial to improve and maintain functional status in patients with ischemic stroke. Further studies are warranted to elucidate the mechanisms underlying the association between abdominal adiposity and functional outcomes after acute ischemic stroke.

### Study strengths and limitations

This study had several strengths. The sample size was large, and clinical variables, including WC, BMI, and insulin action parameters, were prospectively evaluated using standardized methods with few missing values. This study also has some limitations. First, we measured WC in the supine position because of the difficulty in maintaining the upright position after stroke onset, which differs from standard measurements. However, measurements were conducted in the same manner for all patients. Second, multicollinearity is problematic in multivariable models because WC is highly correlated with BMI. However, adjustment for BMI is required to properly assess cardiometabolic risk using WC [11]. In fact, the variance inflation factors of WC were not extremely high (approximately three) in the present study. Finally, as the study patients were enrolled only in the participating hospitals in Japan, the findings should be validated in other settings, including different races or ethnic groups.

## Conclusions

High WC was associated with favorable functional outcomes but not with survival after acute ischemic stroke. This association was independent of body weight or insulin action, and was evident in patients without diabetes. Our findings shed new light on abdominal adiposity as a key factor in understanding the potential role of fat in functional outcomes after stroke.

## Supporting information

S1 Table Fitting of multivariable model for clinical outcomes. AIC: Akaike information criterion, BIC: Bayesian information criterion, MV: multivariable, BMI: body mass index, WC: waist circumference. MV represents multiple variables such as age, sex, hypertension, diabetes mellitus, dyslipidemia, atrial fibrillation, pre-stroke modified Rankin Scale score, previous history of stroke, stroke subtype (cardioembolism, small-vessel occlusion, large-artery atherosclerosis, or others), National Institutes of Health Stroke Scale score on admission, and reperfusion therapy. The model fit was evaluated using the AIC or BIC. Statistical significance was evaluated using the likelihood ratio. *WC or BMI was added to the multivariable model including BMI or WC, respectively.
(PDF)

S2 Table Association between waist circumference and poor functional outcome according to insulin action. MV, multivariable; BMI, body mass index; OR, odds ratio; CI, confidence interval; HOMA-β, homeostatic model assessment of beta cell function; Ph, P for heterogeneity; HOMA-IR, homeostatic model assessment of insulin resistance. Waist circumference was categorized into four groups according to quartiles in females (Q1: ≤74.3 cm, Q2: 74.5–81.8 cm, Q3: 82.0–88.0 cm, and Q4: >89.0 cm) and males (Q1: <78.9 cm, Q2: 79.0–84.9 cm, Q3: 85.0–90.8 cm, and Q4: >91.0 cm). The multivariable model included age, sex, hypertension, diabetes mellitus, dyslipidemia, atrial fibrillation, pre-stroke modified Rankin Scale score, history of stroke, stroke subtype (cardioembolism, small-vessel occlusion, large-artery atherosclerosis, or others), National Institutes of Health Stroke Scale score on admission, and reperfusion therapy. The P value for heterogeneity was evaluated by adding an interaction term of WC categories × subgroup to a multivariable model. *BMI added to multivariable model.
(PDF)

S3 Table Baseline characteristics of patients according to waist circumference in a cohort for discharge outcomes. P_trend_, P for trend; SD, standard deviation; mRS, modified Rankin Scale; NIHSS, National Institutes of Health Stroke Scale; IQR, interquartile range; BMI, body mass index; IRI, immunoreactive insulin; HOMA-β, homeostatic model assessment of beta-cell function; HOMA-IR, homeostatic model assessment of insulin resistance. Data are expressed as mean±SD, n (%), or median (interquartile range). Waist circumference was categorized into four groups according to quartiles in females (Q1: ≤74.3 cm, Q2: 74.5–81.8 cm, Q3: 82.0–88.8 cm, and Q4: ≥89.0 cm) and males (Q1: ≤78.9 cm, Q2: 79.0–84.9 cm, Q3: 85.0–90.8 cm, and Q4: ≥91.0 cm).
(PDF)

S4 Table Association between waist circumference and clinical outcomes at discharge. MV: multivariable, BMI: body mass index, OR: odds ratio, CI: confidence interval. Waist circumference was categorized into four groups according to quartiles in females (Q1: ≤74.3 cm, Q2: 74.5–81.8 cm, Q3: 82.0–88.8 cm, and Q4: ≥89.0 cm) and males (Q1: ≤78.9 cm, Q2: 79.0–84.9 cm, Q3: 85.0–90.8 cm, and Q4: ≥91.0 cm). The multivariable model included age, sex, hypertension, diabetes mellitus, dyslipidemia, atrial fibrillation, pre-stroke modified Rankin Scale score, history of stroke, stroke subtype (cardioembolism, small-vessel occlusion, large-artery atherosclerosis, or others), National Institutes of Health Stroke Scale score on admission, and reperfusion therapy. *BMI added to multivariable model.
(PDF)

S5 Table Association between waist circumference and functional dependency. MV, multivariable; BMI, body mass index; OR, odds ratio; CI, confidence interval. Waist circumference was categorized into four groups according to quartiles in females (Q1: ≤74.3 cm, Q2: 74.5–81.8 cm, Q3: 82.0–88.8 cm, and Q4: ≥89.0 cm) and males (Q1: ≤78.9 cm, Q2: 79.0–84.9 cm, Q3: 85.0–90.8 cm, and Q4: ≥91.0 cm). The multivariable model included age, sex, hypertension, diabetes mellitus, dyslipidemia, atrial fibrillation, pre-stroke modified Rankin Scale score, history of stroke, stroke subtype (cardioembolism, small-vessel occlusion, large-artery atherosclerosis, or others), National Institutes of Health Stroke Scale score on admission, and reperfusion therapy. *BMI added to multivariable model.
(PDF)

S6 Table Association between waist circumference and functional dependency in consideration of insulin metabolism. MV, multivariable; BMI, body mass index; IRI, immunoreactive insulin; HOMA-β, homeostatic model assessment of beta cell function; HOMA-IR, homeostatic model assessment for insulin resistance; OR, odds ratio; CI, confidence interval. Waist circumference was categorized into four groups according to quartiles in females (Q1: ≤74.3 cm, Q2: 74.5–81.8 cm, Q3: 82.0–88.8 cm, and Q4: ≥89.0 cm) and males (Q1: ≤78.9 cm, Q2: 79.0–84.9 cm, Q3: 85.0–90.8 cm, and Q4: ≥91.0 cm). The multivariable model included age, sex, hypertension, diabetes mellitus, dyslipidemia, atrial fibrillation, pre-stroke modified Rankin Scale score, history of stroke, stroke subtype (cardioembolism, small-vessel occlusion, large-artery atherosclerosis, or others), National Institutes of Health Stroke Scale score on admission, and reperfusion therapy. *BMI and IRI, HOMA-β, or HOMA-IR were added to the multivariable model.
(PDF)

S7 Table Fitting of multivariable model for clinical outcomes at discharge. AIC, Akaike information criterion; BIC, Bayesian information criterion; MV, multivariable; BMI, body mass index; WC, waist circumference. MV represents multiple variables such as age, sex, hypertension, diabetes mellitus, dyslipidemia, atrial fibrillation, pre-stroke modified Rankin Scale score, history of stroke, stroke subtype (cardioembolism, small-vessel occlusion, large-artery atherosclerosis, or others), National Institutes of Health Stroke Scale score on admission, and reperfusion therapy. The model fit was evaluated using the AIC or BIC. Statistical significance was evaluated using the likelihood ratio. *WC or BMI was added to the multivariable model including BMI or WC, respectively.
(PDF)

S1 Fig Flow chart of patient selection. (PDF)

S2 Fig Subgroup analysis for the association between WC and functional dependency at 3 months according to age, sex, diabetes mellitus, and obesity. OR, odds ratio; CI, confidence interval; Ph, P for heterogeneity; DM, diabetes mellitus; BMI, body mass index. The association between WC and functional dependency at 3 months is shown separately in subgroups according to age (A, <65 years and ≥65 years), sex (B, females, and males), diabetes mellitus (C, non-diabetes mellitus and diabetes mellitus), and obesity (D, BMI <23 and BMI ≥23). Waist circumference was categorized into four groups according to quartiles in females (Q1: ≤74.3 cm, Q2: 74.5–81.8 cm, Q3: 82.0–88.8 cm, and Q4: ≥89.0 cm) and males (Q1: ≤78.9 cm, Q2: 79.0–84.9 cm, Q3: 85.0–90.8 cm, and Q4: ≥91.0 cm). The multivariable model included age, sex, hypertension, diabetes mellitus, dyslipidemia, atrial fibrillation, pre-stroke modified Rankin Scale score, history of stroke, stroke subtype (cardioembolism, small-vessel occlusion, large-artery atherosclerosis, or others), National Institutes of Health Stroke Scale score on admission, reperfusion therapy, and body mass index. The P value for heterogeneity was evaluated by adding an interaction term of WC categories × subgroup to a multivariable model.
(PDF)
