# Supplementary material for: Association between abdominal adiposity and clinical outcomes in patients with acute ischemic stroke
Source: PLoS One. 2024 Jan 11;19(1):e0296833. doi: 10.1371/journal.pone.0296833 (PMC10783725; doi:10.1371/journal.pone.0296833)
Supplement: S1 Table — AIC: Akaike information criterion, BIC: Bayesian information criterion, MV: multivariable, BMI: body mass index, WC: waist circumference. MV represents multiple variables such as age, sex, hypertension, diabetes mellitus, dyslipidemia, atrial fibrillation, pre-stroke modified Rankin Scale score, previous history of stroke, stroke subtype (cardioembolism, small-vessel occlusion, large-artery atherosclerosis, or others), National Institutes of Health Stroke Scale score on admission, and reperfusion therapy. The model fit was evaluated using the AIC or BIC. Statistical significance was evaluated using the likelihood ratio. *WC or BMI was added to the multivariable model including BMI or WC, respectively. (PDF) [file pone.0296833.s001.pdf]

S1 Table. Fitting of multivariable model for clinical outcomes

	AIC	BIC	P
Poor functional outcome			
MV and BMI	11371	11482	
MV, BMI, and WC*	11363	11495	0.002
Death			
MV and BMI	1920	2030	
MV, BMI, and WC*	1923	2056	0.57
Poor functional outcome			
MV and WC	11367	11492	
MV, WC, and BMI*	11363	11495	0.02
Death			
MV and WC	1940	2065	
MV, WC, and BMI*	1923	2056	<0.001

AIC: Akaike information criterion, BIC: Bayesian information criterion, MV: multivariable, BMI: body mass index, WC: waist circumference.

MV represents multiple variables such as age, sex, hypertension, diabetes mellitus, dyslipidemia, atrial fibrillation, pre-stroke modified Rankin Scale score, previous history of stroke, stroke subtype (cardioembolism, small-vessel occlusion, large-artery atherosclerosis, or others), National Institutes of Health Stroke Scale score on admission, and reperfusion therapy. The model fit was evaluated using the AIC or BIC. Statistical significance was evaluated using the likelihood ratio.

*WC or BMI was added to the multivariable model including BMI or WC, respectively.
