# Supplementary material for: Association between abdominal adiposity and clinical outcomes in patients with acute ischemic stroke
Source: PLoS One. 2024 Jan 11;19(1):e0296833. doi: 10.1371/journal.pone.0296833 (PMC10783725; doi:10.1371/journal.pone.0296833)
Supplement: S2 Table — MV, multivariable; BMI, body mass index; OR, odds ratio; CI, confidence interval; HOMA-β, homeostatic model assessment of beta cell function; Ph, P for heterogeneity; HOMA-IR, homeostatic model assessment of insulin resistance. Waist circumference was categorized into four groups according to quartiles in females (Q1: ≤74.3 cm, Q2: 74.5–81.8 cm, Q3: 82.0–88.0 cm, and Q4: >89.0 cm) and males (Q1: <78.9 cm, Q2: 79.0–84.9 cm, Q3: 85.0–90.8 cm, and Q4: >91.0 cm). The multivariable model included age, sex, hypertension, diabetes mellitus, dyslipidemia, atrial fibrillation, pre-stroke modified Rankin Scale score, history of stroke, stroke subtype (cardioembolism, small-vessel occlusion, large-artery atherosclerosis, or others), National Institutes of Health Stroke Scale score on admission, and reperfusion therapy. The P value for heterogeneity was evaluated by adding an interaction term of WC categories × subgroup to a multivariable model. *BMI added to multivariable model. (PDF) [file pone.0296833.s002.pdf]

S2 Table. Association between waist circumference and poor functional outcome according to insulin action

		Age and sex-adjusted					MV and BMI-adjusted*				
	Events, n (%)	OR	(95% CI)	P	P for trend	Ph	OR	(95% CI)	P	P for trend	Ph
HOMA-β ≤30											
Q1, n=385	191 (49.6)	1.00	(reference)		0.006		1.00	(reference)		0.90	
Q2, n=282	107 (37.9)	0.67	(0.48–0.94)	0.02			0.86	(0.56–1.31)	0.47		
Q3, n=188	65 (34.6)	0.57	(0.38–0.84)	0.005			0.78	(0.46–1.33)	0.37		
Q4, n=136	50 (36.8)	0.72	(0.46–1.11)	0.14			1.09	(0.57–2.08)	0.79		
HOMA-β >30						0.92					0.99
Q1, n=1611	609 (37.8)	1.00	(reference)		<0.001		1.00	(reference)		0.83	
Q2, n=1808	526 (29.1)	0.71	(0.61–0.82)	<0.001			0.81	(0.68–0.97)	0.02		
Q3, n=1955	508 (26.0)	0.62	(0.54–0.72)	<0.001			0.73	(0.60–0.89)	0.002		
Q4, n=1971	500 (25.4)	0.64	(0.55–0.74)	<0.001			0.80	(0.63–1.01)	0.07		
HOMA-IR <2.5											
Q1, n=1715	650 (37.9)	1.00	(reference)		<0.001		1.00	(reference)		0.24	
Q2, n=1697	476 (28.0)	0.67	(0.57–0.77)	<0.001			0.82	(0.68–0.98)	0.03		
Q3, n=1523	380 (25.0)	0.58	(0.50–0.68)	<0.001			0.79	(0.64–0.97)	0.03		
Q4, n=1187	287 (24.2)	0.58	(0.49–0.68)	<0.001			0.89	(0.69–1.16)	0.41		
HOMA-IR ≥2.5						0.23					0.66
Q1, n=282	150 (53.2)	1.00	(reference)		<0.001		1.00	(reference)		0.74	
Q2, n=393	157 (39.9)	0.65	(0.47–0.89)	0.008			0.77	(0.52–1.14)	0.19		
Q3, n=623	195 (31.3)	0.45	(0.33–0.60)	<0.001			0.56	(0.38–0.84)	0.005		
Q4, n=922	265 (28.7)	0.43	(0.32–0.57)	<0.001			0.55	(0.35–0.87)	0.01		

MV, multivariable; BMI, body mass index; OR, odds ratio; CI, confidence interval; HOMA-β, homeostatic model assessment of beta cell function; Ph, P for heterogeneity; HOMA-IR, homeostatic model assessment of insulin resistance.
Waist circumference was categorized into four groups according to quartiles in females (Q1: ≤74.3 cm, Q2: 74.5–81.8 cm, Q3: 82.0–88.0 cm, and Q4: >89.0 cm) and males (Q1: <78.9 cm, Q2: 79.0–84.9 cm, Q3: 85.0–90.8 cm, and Q4: >91.0 cm). The multivariable model included age, sex, hypertension, diabetes mellitus, dyslipidemia, atrial fibrillation, pre-stroke modified Rankin Scale score, history of stroke, stroke subtype (cardioembolism, small-vessel occlusion, large-artery atherosclerosis, or others), National Institutes of Health Stroke Scale score on admission, and reperfusion therapy. The P value for heterogeneity was evaluated by adding an interaction term of WC categories × subgroup to a multivariable model. *BMI added to multivariable model.
