# Supplementary material for: Association between abdominal adiposity and clinical outcomes in patients with acute ischemic stroke
Source: PLoS One. 2024 Jan 11;19(1):e0296833. doi: 10.1371/journal.pone.0296833 (PMC10783725; doi:10.1371/journal.pone.0296833)
Supplement: S3 Table — Ptrend, P for trend; SD, standard deviation; mRS, modified Rankin Scale; NIHSS, National Institutes of Health Stroke Scale; IQR, interquartile range; BMI, body mass index; IRI, immunoreactive insulin; HOMA-β, homeostatic model assessment of beta-cell function; HOMA-IR, homeostatic model assessment of insulin resistance. Data are expressed as mean±SD, n (%), or median (interquartile range). Waist circumference was categorized into four groups according to quartiles in females (Q1: ≤74.3 cm, Q2: 74.5–81.8 cm, Q3: 82.0–88.8 cm, and Q4: ≥89.0 cm) and males (Q1: ≤78.9 cm, Q2: 79.0–84.9 cm, Q3: 85.0–90.8 cm, and Q4: ≥91.0 cm). (PDF) [file pone.0296833.s003.pdf]

S3 Table. Baseline characteristics of patients according to waist circumference in a cohort for discharge outcomes

	Q1, n=2797	Q2, n=2938	Q3, n=3065	Q4, n=3189	P	P_{trend}
Age, y	72±13	71±12	70±11	68±12	<0.001	<0.001
Males	1719 (61.5)	1891 (64.4)	1990 (65.0)	2058 (64.5)	0.02	0.02
Risk factors						
Hypertension	1966 (70.3)	2279 (77.6)	2556 (83.4)	2793 (87.6)	<0.001	<0.001
Diabetes mellitus	597 (21.3)	836 (28.5)	967 (31.5)	1266 (39.7)	<0.001	<0.001
Dyslipidemia	1157 (41.4)	1613 (54.9)	1908 (62.3)	2211 (69.3)	<0.001	<0.001
Atrial fibrillation	758 (27.1)	622 (21.2)	607 (19.8)	616 (19.3)	<0.001	<0.001
Pre-stroke mRS 1	394 (14.1)	353 (12.0)	368 (12.0)	345 (10.8)	0.002	<0.001
Previous stroke	399 (14.3)	463 (15.8)	507 (16.5)	484 (15.2)	0.10	0.27
Stroke subtype						
Cardioembolism	711 (25.4)	541 (18.4)	528 (17.2)	504 (15.8)	<0.001	<0.001
Small-vessel occlusion	707 (25.3)	915 (31.1)	935 (30.5)	1021 (32.0)	<0.001	<0.001
Large-artery atherosclerosis	394 (14.1)	472 (16.1)	510 (16.6)	544 (17.1)	0.01	0.002
Unclassified	985 (35.2)	1010 (34.4)	1092 (35.6)	1120 (35.1)	0.79	0.80
Baseline NIHSS score	3 (1–6)	2 (1–5)	2 (1–4)	2 (1–4)	<0.001	<0.001
Reperfusion therapy	343 (12.3)	306 (10.4)	315 (10.3)	312 (9.8)	0.01	0.003
BMI	19.7±2.3	22.1±2.0	23.8±2.1	27.0±3.4	<0.001	<0.001
Insulin action						
IRI, mU/L	4.5 (3.0–6.9)	5.5 (3.8–8.4)	6.7 (4.6–10.2)	8.7 (5.8–12.7)	<0.001	<0.001
HOMA-β	46 (29–70)	54 (33–82)	61 (38–94)	70 (43–109)	<0.001	<0.001
HOMA-IR	1.1 (0.7–1.9)	1.4 (1.0–2.3)	1.8 (1.1–2.9)	2.3 (1.5–3.7)	<0.001	<0.001

Ptrend, P for trend; SD, standard deviation; mRS, modified Rankin Scale; NIHSS, National Institutes of Health Stroke Scale; IQR, interquartile range; BMI, body mass index; IRI, immunoreactive insulin; HOMA-β, homeostatic model assessment of beta-cell function; HOMA-IR, homeostatic model assessment of insulin resistance.

Data are expressed as mean±SD, n (%), or median (interquartile range). Waist circumference was categorized into four groups according to quartiles in females (Q1: ≤74.3 cm, Q2: 74.5–81.8 cm, Q3: 82.0–88.8 cm, and Q4: ≥89.0 cm) and males (Q1: ≤78.9 cm, Q2: 79.0–84.9 cm, Q3: 85.0–90.8 cm, and Q4: ≥91.0 cm).
