# Supplementary material for: Association between abdominal adiposity and clinical outcomes in patients with acute ischemic stroke
Source: PLoS One. 2024 Jan 11;19(1):e0296833. doi: 10.1371/journal.pone.0296833 (PMC10783725; doi:10.1371/journal.pone.0296833)
Supplement: S4 Table — MV: multivariable, BMI: body mass index, OR: odds ratio, CI: confidence interval. Waist circumference was categorized into four groups according to quartiles in females (Q1: ≤74.3 cm, Q2: 74.5–81.8 cm, Q3: 82.0–88.8 cm, and Q4: ≥89.0 cm) and males (Q1: ≤78.9 cm, Q2: 79.0–84.9 cm, Q3: 85.0–90.8 cm, and Q4: ≥91.0 cm). The multivariable model included age, sex, hypertension, diabetes mellitus, dyslipidemia, atrial fibrillation, pre-stroke modified Rankin Scale score, history of stroke, stroke subtype (cardioembolism, small-vessel occlusion, large-artery atherosclerosis, or others), National Institutes of Health Stroke Scale score on admission, and reperfusion therapy. *BMI added to multivariable model. (PDF) [file pone.0296833.s004.pdf]

S4 Table. Association between waist circumference and clinical outcomes at discharge

		Age and sex-adjusted			MV-adjusted			MV and BMI-adjusted*		
	Events, n (%)	OR	(95% CI)	P	OR	(95% CI)	P	OR	(95% CI)	P
Poor functional outcome										
Q1, n=2797	1297 (46.4)	1.00	(reference)		1.00	(reference)		1.00	(reference)	
Q2, n=2938	1134 (38.6)	0.76	(0.69–0.85)	<0.001	0.81	(0.71–0.92)	0.001	0.83	(0.73–0.95)	0.006
Q3, n=3065	1089 (35.5)	0.68	(0.61–0.76)	<0.001	0.73	(0.64–0.83)	<0.001	0.76	(0.66–0.88)	<0.001
Q4, n=3189	1118 (35.1)	0.72	(0.64–0.80)	<0.001	0.75	(0.66–0.85)	<0.001	0.81	(0.68–0.96)	0.02
P for trend				<0.001			<0.001			0.29
Death										
Q1, n=2797	48 (1.7)	1.00	(reference)		1.00	(reference)		1.00	(reference)	
Q2, n=2938	29 (1.0)	0.64	(0.40–1.02)	0.06	0.75	(0.46–1.22)	0.24	0.99	(0.58–1.69)	0.96
Q3, n=3065	23 (0.8)	0.51	(0.31–0.85)	0.01	0.62	(0.36–1.06)	0.08	0.99	(0.52–1.88)	0.98
Q4, n=3189	16 (0.5)	0.38	(0.21–0.67)	0.001	0.50	(0.27–0.91)	0.02	1.08	(0.47–2.47)	0.86
P for trend				<0.001			0.02			0.55

MV: multivariable, BMI: body mass index, OR: odds ratio, CI: confidence interval.

Waist circumference was categorized into four groups according to quartiles in females (Q1: ≤74.3 cm, Q2: 74.5–81.8 cm, Q3: 82.0–88.8 cm, and Q4: ≥89.0 cm) and males (Q1: ≤78.9 cm, Q2: 79.0–84.9 cm, Q3: 85.0–90.8 cm, and Q4: ≥91.0 cm). The multivariable model included age, sex, hypertension, diabetes mellitus, dyslipidemia, atrial fibrillation, pre-stroke modified Rankin Scale score, history of stroke, stroke subtype (cardioembolism, small-vessel occlusion, large-artery atherosclerosis, or others), National Institutes of Health Stroke Scale score on admission, and reperfusion therapy.

*BMI added to multivariable model.
