# Supplementary material for: Association between abdominal adiposity and clinical outcomes in patients with acute ischemic stroke
Source: PLoS One. 2024 Jan 11;19(1):e0296833. doi: 10.1371/journal.pone.0296833 (PMC10783725; doi:10.1371/journal.pone.0296833)
Supplement: S5 Table — MV, multivariable; BMI, body mass index; OR, odds ratio; CI, confidence interval. Waist circumference was categorized into four groups according to quartiles in females (Q1: ≤74.3 cm, Q2: 74.5–81.8 cm, Q3: 82.0–88.8 cm, and Q4: ≥89.0 cm) and males (Q1: ≤78.9 cm, Q2: 79.0–84.9 cm, Q3: 85.0–90.8 cm, and Q4: ≥91.0 cm). The multivariable model included age, sex, hypertension, diabetes mellitus, dyslipidemia, atrial fibrillation, pre-stroke modified Rankin Scale score, history of stroke, stroke subtype (cardioembolism, small-vessel occlusion, large-artery atherosclerosis, or others), National Institutes of Health Stroke Scale score on admission, and reperfusion therapy. *BMI added to multivariable model. (PDF) [file pone.0296833.s005.pdf]

S5 Table. Association between waist circumference and functional dependency

		Age and sex-adjusted			MV-adjusted			MV and BMI-adjusted*		
	Events, n (%)	OR	(95% CI)	P	OR	(95% CI)	P	OR	(95% CI)	P
At discharge										
Q1, n=2749	1249 (45.4)	1.00	(reference)		1.00	(reference)		1.00	(reference)	
Q2, n=2909	1105 (38.0)	0.77	(0.69–0.86)	<0.001	0.82	(0.72–0.93)	0.002	0.84	(0.73–0.95)	0.008
Q3, n=3042	1066 (35.0)	0.69	(0.62–0.77)	<0.001	0.73	(0.65–0.83)	<0.001	0.76	(0.66–0.88)	<0.001
Q4, n=3173	1102 (34.7)	0.73	(0.66–0.81)	<0.001	0.75	(0.66–0.86)	<0.001	0.81	(0.68–0.96)	0.02
P for trend				<0.001			<0.001			0.27
At 3 months										
Q1, n=2639	1061 (40.2)	1.00	(reference)		1.00	(reference)		1.00	(reference)	
Q2, n=2820	900 (31.9)	0.74	(0.66–0.83)	<0.001	0.78	(0.68–0.89)	<0.001	0.81	(0.71–0.93)	0.004
Q3, n=2961	876 (29.6)	0.67	(0.60–0.76)	<0.001	0.71	(0.62–0.82)	<0.001	0.77	(0.66–0.89)	0.001
Q4, n=3091	892 (28.9)	0.70	(0.63–0.79)	<0.001	0.73	(0.64–0.83)	<0.001	0.83	(0.69–1.00)	0.045
P for trend				<0.001			<0.001			0.49

MV, multivariable; BMI, body mass index; OR, odds ratio; CI, confidence interval.

Waist circumference was categorized into four groups according to quartiles in females (Q1: ≤74.3 cm, Q2: 74.5–81.8 cm, Q3: 82.0–88.8 cm, and Q4: ≥89.0 cm) and males (Q1: ≤78.9 cm, Q2: 79.0–84.9 cm, Q3: 85.0–90.8 cm, and Q4: ≥91.0 cm). The multivariable model included age, sex, hypertension, diabetes mellitus, dyslipidemia, atrial fibrillation, pre-stroke modified Rankin Scale score, history of stroke, stroke subtype (cardioembolism, small-vessel occlusion, large-artery atherosclerosis, or others), National Institutes of Health Stroke Scale score on admission, and reperfusion therapy.

*BMI added to multivariable model.
