# Supplementary material for: Association between abdominal adiposity and clinical outcomes in patients with acute ischemic stroke
Source: PLoS One. 2024 Jan 11;19(1):e0296833. doi: 10.1371/journal.pone.0296833 (PMC10783725; doi:10.1371/journal.pone.0296833)
Supplement: S6 Table — MV, multivariable; BMI, body mass index; IRI, immunoreactive insulin; HOMA-β, homeostatic model assessment of beta cell function; HOMA-IR, homeostatic model assessment for insulin resistance; OR, odds ratio; CI, confidence interval. Waist circumference was categorized into four groups according to quartiles in females (Q1: ≤74.3 cm, Q2: 74.5–81.8 cm, Q3: 82.0–88.8 cm, and Q4: ≥89.0 cm) and males (Q1: ≤78.9 cm, Q2: 79.0–84.9 cm, Q3: 85.0–90.8 cm, and Q4: ≥91.0 cm). The multivariable model included age, sex, hypertension, diabetes mellitus, dyslipidemia, atrial fibrillation, pre-stroke modified Rankin Scale score, history of stroke, stroke subtype (cardioembolism, small-vessel occlusion, large-artery atherosclerosis, or others), National Institutes of Health Stroke Scale score on admission, and reperfusion therapy. *BMI and IRI, HOMA-β, or HOMA-IR were added to the multivariable model. (PDF) [file pone.0296833.s006.pdf]

S6 Table. Association between waist circumference and functional dependency in consideration of insulin metabolism

		MV, BMI, and IRI-adjusted*			MV, BMI, and HOMA-β-adjusted*			MV, BMI, and HOMA-IR-adjusted*		
	Events, n (%)	OR	(95% CI)	P	OR	(95% CI)	P	OR	(95% CI)	P
At discharge										
Q1, n=2366	1020 (43.1)	1.00	(reference)		1.00	(reference)		1.00	(reference)	
Q2, n=2428	835 (34.4)	0.81	(0.70–0.95)	0.01	0.81	(0.69–0.95)	0.01	0.81	(0.69–0.95)	0.009
Q3, n=2541	806 (31.7)	0.73	(0.61–0.86)	<0.001	0.74	(0.62–0.88)	0.001	0.73	(0.61–0.87)	<0.001
Q4, n=2503	766 (30.6)	0.77	(0.63–0.96)	0.02	0.78	(0.63–0.97)	0.03	0.77	(0.63–0.96)	0.02
P for trend				0.46			0.56			0.45
At 3 months										
Q1, n=2276	874 (38.4)	1.00	(reference)		1.00	(reference)		1.00	(reference)	
Q2, n=2358	684 (29.0)	0.81	(0.68–0.95)	0.01	0.80	(0.68–0.94)	0.008	0.80	(0.68–0.94)	0.007
Q3, n=2472	659 (26.7)	0.74	(0.61–0.88)	0.001	0.74	(0.61–0.88)	0.001	0.73	(0.61–0.88)	0.001
Q4, n=2442	625 (25.6)	0.80	(0.64–1.00)	0.05	0.81	(0.64–1.01)	0.06	0.79	(0.63–0.99)	0.045
P for trend				0.58			0.66			0.54

MV, multivariable; BMI, body mass index; IRI, immunoreactive insulin; HOMA-β, homeostatic model assessment of beta cell function; HOMA-IR, homeostatic model assessment for insulin resistance; OR, odds ratio; CI, confidence interval.

Waist circumference was categorized into four groups according to quartiles in females (Q1: ≤74.3 cm, Q2: 74.5–81.8 cm, Q3: 82.0–88.8 cm, and Q4: ≥89.0 cm) and males (Q1: ≤78.9 cm, Q2: 79.0–84.9 cm, Q3: 85.0–90.8 cm, and Q4: ≥91.0 cm). The multivariable model included age, sex, hypertension, diabetes mellitus, dyslipidemia, atrial fibrillation, pre-stroke modified Rankin Scale score, history of stroke, stroke subtype (cardioembolism, small-vessel occlusion, large-artery atherosclerosis, or others), National Institutes of Health Stroke Scale score on admission, and reperfusion therapy.

*BMI and IRI, HOMA-β, or HOMA-IR were added to the multivariable model.
