# Supplementary material for: Association between abdominal adiposity and clinical outcomes in patients with acute ischemic stroke
Source: PLoS One. 2024 Jan 11;19(1):e0296833. doi: 10.1371/journal.pone.0296833 (PMC10783725; doi:10.1371/journal.pone.0296833)
Supplement: S7 Table — AIC, Akaike information criterion; BIC, Bayesian information criterion; MV, multivariable; BMI, body mass index; WC, waist circumference. MV represents multiple variables such as age, sex, hypertension, diabetes mellitus, dyslipidemia, atrial fibrillation, pre-stroke modified Rankin Scale score, history of stroke, stroke subtype (cardioembolism, small-vessel occlusion, large-artery atherosclerosis, or others), National Institutes of Health Stroke Scale score on admission, and reperfusion therapy. The model fit was evaluated using the AIC or BIC. Statistical significance was evaluated using the likelihood ratio. *WC or BMI was added to the multivariable model including BMI or WC, respectively. (PDF) [file pone.0296833.s007.pdf]

S7 Table. Fitting of multivariable model for clinical outcomes at discharge

	AIC	BIC	P
Poor functional outcome			
MV and BMI	12124	12235	
MV, BMI, and WC*	12117	12250	0.003
Functional dependency			
MV and BMI	12059	12170	
MV, BMI, and WC*	12052	12184	0.003
Death			
MV and BMI	1015	1126	
MV, BMI, and WC*	1021	1154	0.99
Poor functional outcome			
MV and WC	12116	12242	
MV, WC, and BMI*	12117	12250	0.22
Functional dependency			
MV and WC	12051	12176	
MV, WC, and BMI*	12052	12184	0.26
Death			
MV and WC	1026	1152	
MV, WC, and BMI*	1021	1154	0.008

AIC, Akaike information criterion; BIC, Bayesian information criterion; MV, multivariable; BMI, body mass index; WC, waist circumference.
MV represents multiple variables such as age, sex, hypertension, diabetes mellitus, dyslipidemia, atrial fibrillation, pre-stroke modified Rankin Scale score, history of stroke, stroke subtype (cardioembolism, small-vessel occlusion, large-artery atherosclerosis, or others), National Institutes of Health Stroke Scale score on admission, and reperfusion therapy. The model fit was evaluated using the AIC or BIC. Statistical significance was evaluated using the likelihood ratio.
*WC or BMI was added to the multivariable model including BMI or WC, respectively.
