# Supplementary figures and images for: Association between abdominal adiposity and clinical outcomes in patients with acute ischemic stroke
Source: PLoS One. 2024 Jan 11;19(1):e0296833. doi: 10.1371/journal.pone.0296833 (PMC10783725; doi:10.1371/journal.pone.0296833)

**S1 Fig. Flow chart of patient selection**

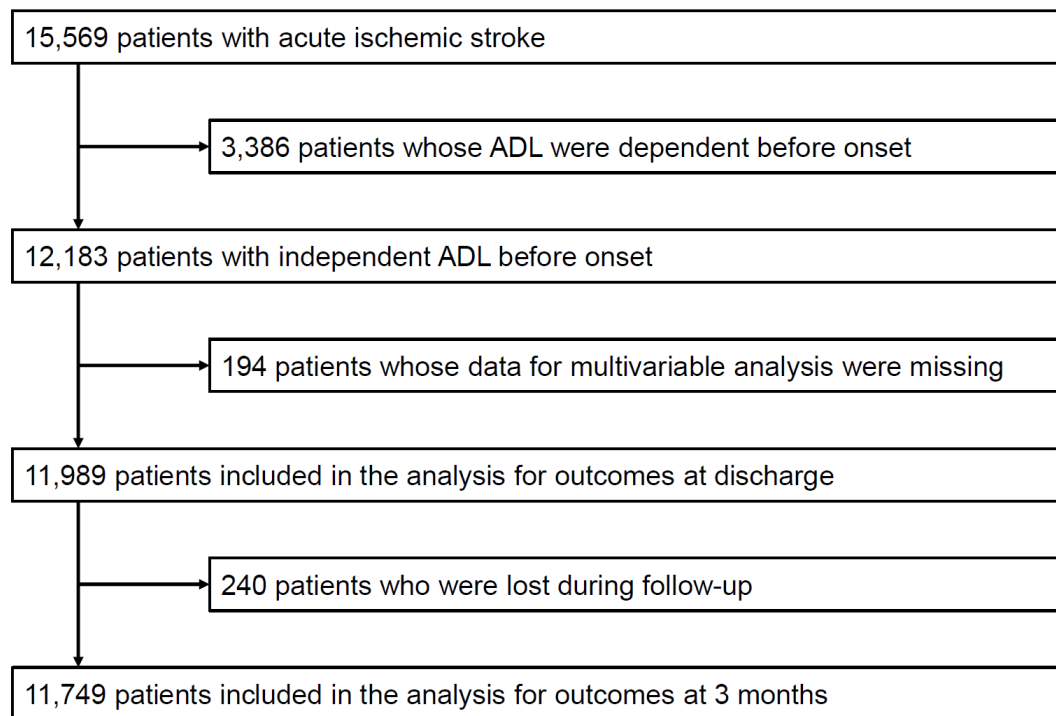

Supplement: S1 Fig — (PDF) [file pone.0296833.s008.pdf]
