# Supplementary material for: Association between abdominal adiposity and clinical outcomes in patients with acute ischemic stroke
Source: PLoS One. 2024 Jan 11;19(1):e0296833. doi: 10.1371/journal.pone.0296833 (PMC10783725; doi:10.1371/journal.pone.0296833)
Supplement: S2 Fig — OR, odds ratio; CI, confidence interval; Ph, P for heterogeneity; DM, diabetes mellitus; BMI, body mass index. The association between WC and functional dependency at 3 months is shown separately in subgroups according to age (A, <65 years and ≥65 years), sex (B, females, and males), diabetes mellitus (C, non-diabetes mellitus and diabetes mellitus), and obesity (D, BMI <23 and BMI ≥23). Waist circumference was categorized into four groups according to quartiles in females (Q1: ≤74.3 cm, Q2: 74.5–81.8 cm, Q3: 82.0–88.8 cm, and Q4: ≥89.0 cm) and males (Q1: ≤78.9 cm, Q2: 79.0–84.9 cm, Q3: 85.0–90.8 cm, and Q4: ≥91.0 cm). The multivariable model included age, sex, hypertension, diabetes mellitus, dyslipidemia, atrial fibrillation, pre-stroke modified Rankin Scale score, history of stroke, stroke subtype (cardioembolism, small-vessel occlusion, large-artery atherosclerosis, or others), National Institutes of Health Stroke Scale score on admission, reperfusion therapy, and body mass index. The P value for heterogeneity was evaluated by adding an interaction term of WC categories × subgroup to a multivariable model. (PDF) [file pone.0296833.s009.pdf]

S2 Fig. Subgroup analysis for the association between WC and functional dependency at 3 months according to age, sex, diabetes mellitus, and obesity

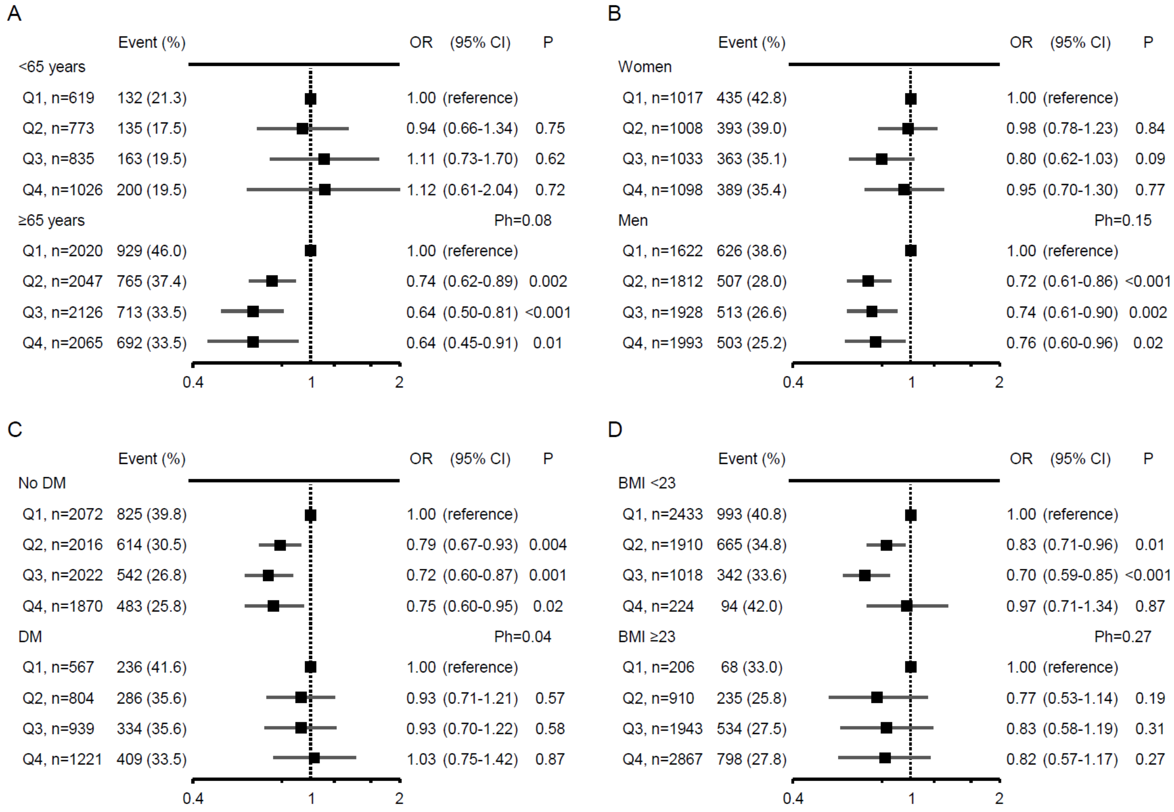

OR, odds ratio; CI, confidence interval; Ph, P for heterogeneity; DM, diabetes mellitus; BMI, body mass index.

The association between WC and functional dependency at 3 months is shown separately in subgroups according to age (A, <65 years and ≥65 years), sex (B, females, and males), diabetes mellitus (C, non-diabetes mellitus and diabetes mellitus), and obesity (D, BMI <23 and BMI ≥23). Waist circumference was categorized into four groups according to quartiles in females (Q1: ≤74.3 cm, Q2: 74.5–81.8 cm, Q3: 82.0–88.8 cm, and Q4: ≥89.0 cm) and males (Q1: ≤78.9 cm, Q2: 79.0–84.9 cm, Q3: 85.0–90.8 cm, and Q4: ≥91.0 cm). The multivariable model included age, sex, hypertension, diabetes mellitus, dyslipidemia, atrial fibrillation, pre-stroke modified Rankin Scale score, history of stroke, stroke subtype (cardioembolism, small-vessel occlusion, large-artery atherosclerosis, or others), National Institutes of Health Stroke Scale score on admission, reperfusion therapy, and body mass index. The P value for heterogeneity was evaluated by adding an interaction term of WC categories × subgroup to a multivariable model.
